# Association of the BB genotype of the ABO gene with the risk of acute myocardial infarction in hospital-based study

**DOI:** 10.12669/pjms.39.1.5905

**Published:** 2023

**Authors:** Farzana Abubakar Yousuf, Iqbal Azam, Asal Khan Tareen, Khawar A Kazmi, Jibran Sualeh Muhammad, Mohammad Perwaiz Iqbal

**Affiliations:** 1 Farzana Abubakar Yousuf, Department of Biological and Biomedical Sciences, Aga Khan University, Karachi, Pakistan; 2 Iqbal Azam, Department of Community Health Sciences, Aga Khan University, Karachi, Pakistan; 3 Asal Khan Tareen, National Institute of Cardiovascular Diseases (NICVD), Karachi, Pakistan; 4 Khawar A Kazmi, National Institute of Cardiovascular Diseases (NICVD), Karachi, Pakistan; 5 Jibran Sualeh Muhammad, Basic Medical Sciences, College of Medicine, University of Sharjah, United Arab Emirates. Department of Biological and Biomedical Sciences, Aga Khan University, Karachi, Pakistan; 6 Mohammad Perwaiz Iqbal, Department of Life Sciences, University of Management and Technology, Lahore-54770, Pakistan. Pakistan Academy of Sciences, Islamabad, Pakistan. Department of Biological and Biomedical Sciences, Aga Khan University, Karachi, Pakistan

**Keywords:** ABO blood groups, ABO genotypes, Acute myocardial infarction, Association study, Pakistani population

## Abstract

**Objectives:**

The ABO gene locus has been identified to be associated with myocardial infarction in patients with coronary heart disease. The primary focus of this hospital-based study was to explore the relationship of ABO blood groups and ABO genotypes with acute myocardial infarction (AMI) in Karachi, Pakistan.

**Methods:**

In a comparative cross-sectional study, an equal number of adult AMI patients and healthy controls (n=275 in each group; age range 30-70 years, both males and females) were recruited from the Aga Khan University and NICVD, Karachi, with informed consent. The blood samples were analyzed for ABO blood groups and other biomarkers. PCR followed by RFLP techniques were employed for determining the ABO genotypes. Multinomial regression was used to evaluate the association of genotypes with the risk of AMI.

**Results:**

Thirteen different combinations of ABO genotypes were observed while the O^2^O^2^ and A^2^A^2^ genotypes were not detected. No significant association based on the distribution of blood groups A, B, O and AB among AMI patients and healthy individuals was observed. The odds of AMI were 3.32 times in subjects with BB genotype as compared to subjects with OO genotypes after adjustment of age, gender, body mass index, heart rate, total cholesterol, and waist circumference [AOR (95% CI) =3.32 (1.36-8.08), p-value =0.008].

**Conclusion:**

Our hospital-based study indicates that ABO genotype BB was significantly associated with the risk of AMI. This harmful effect of the BB genotype could have a possible relationship with AMI’s development in the Pakistani population.

## INTRODUCTION

Coronary artery disease (CAD) accounts for approximately 20.28% of all deaths in Pakistan and this has ranked Pakistan as number 18 in the world as the age-adjusted death rate was 237.98 per 100,000 of population.^1^ A paper by leading cardiologists of the country highlighted the profile of 1,400 acute myocardial infarction (AMI) patients and showed that 68% of them were males and the mean age of all of them at presentation was 52.2 years, almost ten years earlier than the Western population.^2^ This indicates that genetic factor could also be contributing to high rates of CAD in Pakistan.

The four main phenotypes of ABO blood groups i.e. A, B, O and AB were differentiated based on the type of antigen present on the erythrocytes. These blood groups have been reported to be associated with various diseases, specifically CAD and venous thromboembolism.^3^ A systematic review by Chen et al. has shown an association of blood group A and non-O blood groups with increased risk of CAD.^4^ A recent systematic review on frequency distribution of blood groups in Pakistan reported that nearly two-third of the Pakistani population belonged to that non-O groups (B=33.3%, A=23.99% and AB=9.74%), therefore they are at increased risk of developing CAD.^5^

Genotype-wise, there are five ABO alleles (A^1^, A^2^, B, O^1^, O^2^) differing from each other due to substitution or deletion at various nucleotide positions of the ABO gene. Fifteen ABO genotypes have been identified.^6^ Whereas a few studies have been carried out on the association of ABO blood groups with CAD, there are hardly any published reports from South Asia on the relationship between ABO genotypes and AMI, except one recent report from India.^7^ Therefore, the present study aimed to explore the relationship of blood groups and ABO genotypes with AMI in a Pakistani population in Karachi and determine any influence of well-known risk factors for CAD on the relationship of ABO genotypes with AMI in this population.

## METHODS

### Study design, recruitment of subjects, sample size and ethical approval:

A comparative cross-sectional study included both AMI patients (275) and normal healthy controls (275). Assuming a prevalence of B allele to be 39% and O allele 27%, a minimum sample size of 240 patients and 240 healthy subjects would achieve 80% power to detect an odds ratio of at least 2 at 5% level of significance (www.ClinCalc.com/ststs/samplesize.aspx). Therefore, 275 AMI patients were recruited from the two hospitals i.e. National Institute of Cardiovascular Diseases (NICVD), Karachi, and the Aga Khan University Hospital (AKUH), Karachi, from February 2015 to September 2016. The AMI patients (age 30-70 years) were diagnosed as per WHO clinical practice guidelines suggestive of ischemic heart disease, electrocardiogram (ECG) changes of myocardial injury, and higher levels of troponin-I. They were evaluated for hypercholesterolemia, hypertension, obesity, and type 2 diabetes mellitus.

Similarly, 275 apparently healthy people with no history of CAD (age 30-70 years) were enrolled based on physical and clinical examination with appropriate biochemical markers to function as controls. This study did not include both patients and controls with a history of epilepsy, malabsorption syndrome, liver disease, tuberculosis, uremia, or cancer. Pregnant women and those using oral contraceptives were also excluded. Demographic and clinical characteristics of study participants were obtained. The data include variables like gender (male/female), age, ethnicity, waist circumference, body weight (kg), height (m) and blood pressure (BP). Body mass index (BMI, kg/m^2^) was calculated using body weight and height. The total duration of study was from February 2015 to May 20, 2020.

The study was approved by the Ethics Review Committees (ERCs) of AKU (Ref#-3048-BBS-ERC-14) and NICVD, Karachi (Ref.-#-ERC-10/2015). Participants were included with informed consent.

### Blood Sampling and Measurement of Biomarkers:

Non-fasting blood samples of AMI patients and fasting blood samples of healthy controls (10ml) were collected into the vacutainer tubes containing dipotassium ethylenediaminetetraacetic acid and another set of tubes for serum. White blood cells were used for DNA extraction, while whole blood was used to determine blood groups by agglutination reaction. The determination of total serum cholesterol, and serum glucose was carried out using kits obtained from Roche Diagnostics, USA.

### Genotyping of the ABO Gene:

Genomic DNA was extracted and purified by employing a standard protocol.^8^ The ABO gene (exons 6 and 7) fragments were amplified by PCR using the primers described by Olsson and Chester.^6^ Restriction fragment length polymorphism (RFLP) technique was used for genotyping. The *Kpn*I and *Hpa*II (restriction enzymes; New England Biolabs, USA) were used for the digestion of the amplified products,^6^,^9^ and digested fragments were visualized by agarose gel electrophoresis.

### Statistical Analysis:

Statistical analysis of data was carried out on SPSS version 19.0 for Windows. Cross tabulation (using percentage and count) for gender and ABO genotype with AMI status (AMI and normal) were obtained. Means and standard deviations of continuous variables by AMI status were compared using independent samples t-test separately for males and females. The associations were observed using chi-square or Fisher Exact test, whichever was appropriate.

The crude and adjusted relationship between ABO genotypes and the associated risk of AMI was explored by using multinomial logistic regression and reported as crude and adjusted Odds Ratios (95% CI). A p value < 0.05 was considered significant.

## RESULTS

The baseline characteristics of normal healthy controls and AMI patients have been shown in Table-IA. There appears a predisposition of the male gender towards AMI compared to the female gender (p < 0.001). Waist circumference was high among female AMI patients than normal healthy females (p = 0.03). The low levels of total cholesterol among patients could be because of being on cholesterol-lowering medications. We chose non-fasting blood from AMI patients due to logistic reasons and knowing that fasting and non-fasting status has no effect on total cholesterol we measured total serum cholesterol in this study.^10^

**Table-IA:** Baseline characteristics of the normal healthy controls and AMI patients in hospital-based Pakistani population.

Variables	Normal Healthy Controls (n =275) n (%)	AMI Patients (n =275) n (%)	p-value *
***Gender***			<0.001
Males	168 (61.1)	223 (81.1)	
Females	107 (38.9)	52 (18.9)	

	*Mean ± SD*	*Mean ± SD*	

***Age(years)***			
Males	40.8± 7.9	53.4 ± 9.2	<0.001
Females	40.4 ± 7.8	55.3 ± 9.4	<0.001
***BMI(kg/m^2^)***			
Males	25.7 ± 3.4	24.8 ± 4.0	0.029
Females	27.2 ± 4.0	27.3 ± 4.9	0.93
***Blood Pressure (mm of Hg) Systolic BP***			
Males	118 ± 11	119 ± 14	0.56
Females	114 ± 8	125 ± 17	<0.001
***Diastolic BP***			
Males	78 ± 8	76 ± 10	0.016
Females	74 ±7	78 ± 8	0.004
***WC (cm)***			
Males	91.4 ± 11.2	93.3 ± 8.8	0.06
Females	89.4 ± 13.1	93.8 ± 9.0	0.03
***TC (mg/dl)***			
Males	174.6 ± 28.7	162.5 ± 42.8	<0.002
Females	172.2 ± 28.5	169.8 ± 52.3	0.699
***FSG (mg/dl)***			
Males	87.9 ± 10.2		<0.005
Females	84.4 ± 10.6		
***RSG (mg/dl)***			
Males		157.4 ± 83.3	<0.001
Females		224.3 ± 87.1	

BMI: body mass index, BP: blood pressure, WC: waist circumference, TC: total cholesterol, FSG: fasting serum glucose, RSG: random serum glucose,

* p-value represents the comparison of percentages in two groups tested by chi-square test and the means were compared using Independent sample t test.

**Fig.1:**
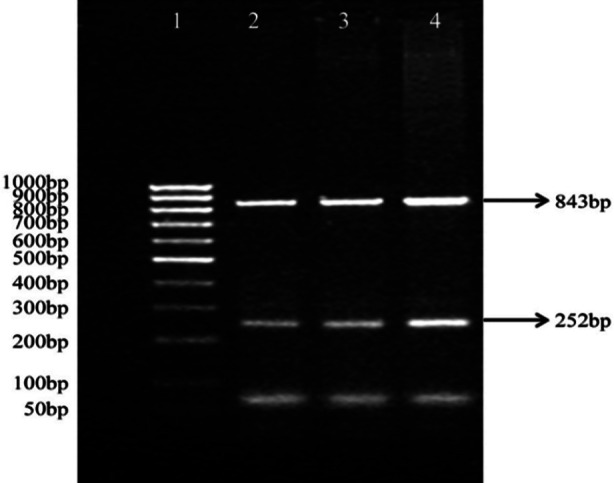
2% agarose gel showing the amplification of ABO gene (exons 6 and 7). The primer pairs mo46/57 which yields 252bp fragment and mo101/71 primer pair which yields 843bp fragment, Lane 1 shows 50-1000bp ladder, Lane 2-4 are the PCR amplification bands.

**Fig.2:**
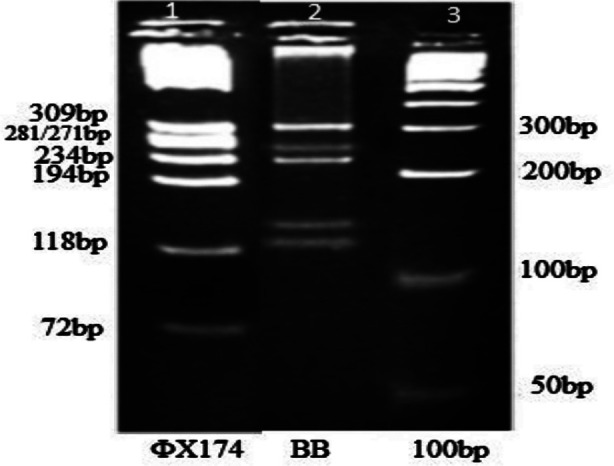
Digestion patterns of the BB genotypes using KpnI and HpaII restriction enzymes separated on a 4% agarose gel Lane 1 shows ΦX174 /HaeIII marker, Lane 2 shows BB genotype pattern having 309bp, 252bp, 223bp, 137bp and 119bp, fragments, Lane 3 shows 100bp marker.

Table-IIB showing the distribution of different types of blood groups by ethnicities indicates that the sampled population had good representation of the four major ethnicities in Karachi. However, because of small proportions of blood groups in these ethnicities these were not included in the final analysis. The frequency of blood group B was highest amongst AMI patients and healthy controls (39.3% vs. 38.5%). The second most predominant blood group among AMI patients was A (26.2%), followed by O (24.0%) and AB (10.5%) blood group. Among the normal healthy controls, the second most widely distributed blood group was O (29.1%), followed by A (23.6%) and then AB (8.7%). However, no significant association in the distribution frequencies of blood groups was observed amongst both groups. Overall, the B blood group was most predominant. Fig.1 is a typical representative gel (2% agarose) showing the amplification of the ABO gene (exons 6 and 7). The primer-pair mo-46/mo-57 yields a 252 bp fragment in exon 6, while primer-pair mo-101/mo-71 yields 843 bp fragment after amplification. These fragments after digestion with *Kpn*I and *Hpa*II would yield fragments of various sizes representing 15 genotypes.^6^,^11^ Fig.2 illustrates the digestion patterns of BB genotypes using *Kpn*I and *Hpa*II restriction enzymes separated on 4% agarose gel. .A total of thirteen genotypes were found in our study subjects, while genotype O^2^O^2^ and A^2^A^2^ were not observed. The frequency distribution of ABO genotypes is shown in Table-II. The BO^1^ genotype was the most prevalent in normal healthy controls (29.8%), while it was 21.5% in the AMI patients (p = 0.031). The prevalence of BB genotype was significantly different between the two groups. (p = 0.004).

**Table-I-B:** Distribution of frequencies of blood groups with respect to ethnicity among healthy controls and AMI patients

Normal Healthy Controls (n =275) n (%)					AMI Patients (n =275) n (%)				

Groups n (%)	AB n (%)	A n (%)	B n (%)	O n (%)	Groups	AB n (%)	A n (%)	B n (%)	O n (% )
Punjabi 37(13.5)	4(1.45)	10(3.63)	12(4.36)	11(4.0)	Punjabi 54(19.6)	4 (1.45)	15(5.45)	23(8.36)	12 (4.36)
Sindhi 16(5.8)	1(0.36)	3(1.09)	7(2.54)	5(1.81)	Sindhi 34(12.4)	6 (2.18)	13(4.72)	6 (2.18)	9 (3.27)
Pathan 12(4.4)	1(0.36)	3(1.09)	6(2.18)	2(0.72)	Pathan 26(9.5)	3(1.09)	6 (2.18)	10(3.63)	7 (2.54)
Urdu Speaking 84(30.5)	7(2.54)	20(7.35)	33(12)	24(8.72)	Urdu Speaking 114(41.5)	14(5.09)	26(9.45)	49(17.81)	25(9.09)
Others 126(45.8)	12(4.36)	28(10.18)	49(17.8)	37(13.45)	Others 47(17.1)	2 (0.72)	12(4.36)	21(7.63)	12 (4.36)

**Table-II:** Distribution of frequencies of ABO genotypes in normal healthy controls and AMI patients in hospital-based Pakistani population

	Controls	Frequency (%)	AMI Patients	Frequency (%)	p-value*
BB	17	6.2	38	13.8	0.004
BO^1^	82	29.8	59	21.5	0.031
BO^2^	8	2.9	12	4.4	0.49
O^1^ O^1^	76	27.6	63	22.9	0.24
O^1^ O^2^	3	1.1	2	0.7	0.99
A^1^B	23	8.4	24	8.7	>0.999
A^2^B	2	0.7	5	1.8	0.45
A^1^ A^1^	6	2.2	7	2.5	>0.999
A^1^ A^2^	2	0.7	7	2.5	0.18
A^1^ O^1^	47	17.1	48	17.5	>0.999
A^1^ O^2^	1	0.4	1	0.4	>0.999
A^2^O^1^	7	2.5	7	2.5	>0.999
A^2^ O^2^	1	0.4	2	0.7	0.99

* p-value was determined using chi-square test or Fisher Exact test.

In order to study the association of BB genotype with AMI, some of the sub-alleles were combined into the common allele such as O^1^and O^2^ into a common “O” allele. After combining sub-alleles present in small numbers, the resulting 6 genotypes were then analyzed by multinomial regression analysis (Table-III). The “OO” genotype was taken as the reference genotype. Analysis revealed that BB genotype was directly associated with the risk of having AMI and odds were 3.32 folds high in subjects with BB genotype compared to OO genotype subjects when the model was adjusted for age, gender, BMI, heart rate, total cholesterol and waist circumference [AOR (95% CI) = 3.32 (1.36-8.08); p = 0.008; Table-III].

**Table-III:** Multinomial logistic regression of the ABO genotypes with the risk of AMI in hospitalbased Pakistani population before and after adjustment for covariates

Genotypes	Crude OR (95% CI)	p-value*
OO (Ref.)	1.0	-
BB	2.72 (1.41, 5.25)	0.003
BO	0.96 (0.61, 1.51)	0.855
AB	1.41 (0.75, 2.64)	0.283
AA	2.13 (0.84, 5.43)	0.111
AO	1.26 (0.77, 2.06)	0.36

*Genotype BB adjusted for covariates*	*Adjusted OR (95% CI)*	*p-value*

Adjusted^2^	3.87 (1.71-8.76)	<0.001
Adjusted^3^	3.94(1.71-9.01)	<0.001
Adjusted^4^	3.88 (1.68-8.94)	<0.001
Adjusted^5^	3.39 (1.41-8.14)	0.006
Adjusted^6^	3.31 (1.36-8.03)	0.008
Adjusted^7^	3.32 (1.36-8.08)	0.008

1 Values are OR (95%CI) using multinomial logistic regression.

2 AOR (95%CI) using multinomial logistic regression while adjusted for age.

3 AOR (95%CI) using multinomial logistic regression while adjusted for age and gender.

4 AOR (95%CI) using multinomial logistic regression while adjusted age, gender and BMI.

5 AOR (95%CI) using multinomial logistic regression while adjusted for age, gender, BMI and heart rate.

6 AOR (95%CI) using multinomial logistic regression while adjusted for age, gender, BMI, heart rate and total cholesterol.

7 AOR (95%CI) using multinomial logistic regression while adjusted for age, gender, BMI, heart rate, total cholesterol, and waist circumference.

## DISCUSSION

Overall, the AMI patients’ data showed a predisposition of males towards AMI (81.1%) as compared to females (18.9%) (p < 0.001). This is in line with the reports from Pakistan which showed that the proportions of male CAD patients were 69.13% and 79.2% among the population in North Punjab and Rawalpindi, respectively.^12^,^13^ Obesity is known to be a risk factor for CAD.^14^ In the present study, females in both groups were found to be obese. This is supported by a recent report indicating that Pakistani women were more obese than men.^15^ Thus they are at higher risk of developing AMI.

The frequency of blood groups distribution differs among various communities and geographical regions all over the world.^16^ Hirschfield showed that ABO blood group frequencies varied widely across human population groups. Blood group A was more predominant amongst Western Europeans, while the B blood group was more prevalent among the Indians.^17^ However, distribution frequencies of blood groups in AMI patients and healthy controls in this study showed that blood group B was most prevalent. Similar results have been reported in studies in Pakistan and India.^18^,^19^

In the present study, no association was observed between AMI patients and blood groups. Similar findings have been reported in an Iranian study showing a lack of association between blood groups and CAD.^20^Thirteen ABO genotypes were identified in this Pakistani population, while O^2^O^2^ and A^2^A^2^were not detected. Similar findings have been reported in an Indian population.^7^

In the present study, the BB (p value = 0.004) and BO^1^ (p value = 0.031) genotypes appeared to have a significant association with AMI, while no significant difference in the frequency distribution of other ABO genotypes was observed between the two groups. The reason could be a very small number of certain ABO alleles. Our findings regarding the moderate prevalence of O^1^O^1^ genotype (22.9%) differed from the Kuwaiti population where the O^1^O^1^ genotype was highly prevalent (47.04%).^11^

The binary logistic regression model revealed that odds of BO^1^ genotype had protective effects towards the risk of AMI. In order to confirm this association, the sub-alleles in small numbers were combined into common alleles and the resulting six genotypes groups were analyzed by multinomial logistic regression with the OO genotype as the reference. Only BB genotype had a significant association with odds of having AMI 3.32 times greater in subjects with BB genotypes as compared to the genotype OO when the regression model was adjusted for age, gender, BMI, heart rate, total cholesterol and waist circumference. This is supported by a recent report from India where BB genotype was also found to be associated with myocardial infarction.^7^ Moreover, a Swedish study also showed 2.7 times the risk of AMI in Swedish patients with B allele.^21^

The mechanisms through which certain genotypes could increase the risk of CAD is unclear; however, various hypotheses have been put forward. For example, certain genotypes could be contributing to the development of thrombosis. Accordingly, the A^1^ and B alleles have a two-fold increased risk for developing deep venous thrombosis. The non-OO genotypes except homozygous A^2^A^2^or A^2^O^1^or A^2^O^2^ have been found to have a higher risk of venous thrombosis than the OO genotype.^22^ According to Wiggins et al., the A, B and AB blood groups have greater than 30% risk for thromboembolic events than blood group O.^23^ All these reports point towards the increased risk of thrombosis in subjects with A, B and AB blood groups.

Another possible mechanism could be increased adhesiveness of platelets in certain ABO blood group subjects. 24 The B blood group subjects had reportedly high platelet distribution width, compared to A and O indicating the activation of platelets and the process of inflammation.^25^

In a study, von Willebrand factor (VWF) *levels were elevated among* subjects with BB genotype.^26^ It is unclear how blood type B affects the VWF levels. However, it has been hypothesized that ABH antigens affect the plasma clearance rates of VWF, which results in elevation of VWF levels in plasma and ultimately increases the risk for myocardial infarction.^21^ It has also been hypothesized that platelet aggregation is supported by the B antigen, hence leading to myocardial infarction.

### Limitations:

One of the limitations of this study was that some of the alleles were found in very small numbers; therefore, their association with AMI could not be investigated.

## CONCLUSION

ABO genotype ‘BB’ was significantly associated with an increased risk of developing AMI in the Pakistani population. The odds of AMI among subjects with BB genotype were 3.32 times high. Further large-scale studies involving major ethnic groups in Pakistan are needed to find conclusive evidence regarding the relationship between blood groups, genotypes and AMI in the adult Pakistani population.
